# The Effect of Intracanal Medicaments on Microleakage of Mineral Trioxide Aggregate Apical Plugs

**DOI:** 10.22037/iej.v12i3.15978

**Published:** 2017

**Authors:** Mohammadreza Nabavizadeh, Fariborz Moazzami, Mohsen Bahmani, Hossein Mirhadi

**Affiliations:** a *Oral and Dental Disease Research Center, Dental School, Shiraz University of Medical Sciences, Shiraz, Iran; *; b *Department of Endodontics, Dental school, Shiraz University of Medical Sciences, Shiraz, Iran*

**Keywords:** Calcium Hydroxide, Double Antibiotic Paste, Endodontic Regeneration, Microleakage, Triple Antibiotic Paste

## Abstract

**Introduction:**

The purpose of this *in vitro* study was to evaluate the effect of calcium hydroxide, double and triple antibiotic paste on the sealing ability of mineral trioxide aggregate (MTA) apical plugs.

**Methods and Materials:**

A total of 90 extracted teeth with single canals were prepared and randomly divided into four experimental groups (*n*=20). Intra-canal medicaments were applied for 3 weeks. MTA was placed through the access opening and condensed to the apical area and then fluid filtration technique was utilized to evaluate sealing ability after 1, 7, 14 and 30 days.

**Results:**

Triple antibiotic paste significantly reduced the sealing ability of MTA plug compared with double antibiotic paste (*P*=0.024) and normal saline (*P*=0.04) groups on day 1. The sealing ability was not different on days 14 and 30 between experimental groups (*P*>0.05).

**Conclusion:**

All medicaments can be used without any long term effect on microleakage.

## Introduction

Endodontic treatment of immature necrotic teeth has always been challenging and an adequate apical seal is not always achievable using conventional endodontic methods [1]. As an alternative clinical approach to apexification and the apical barrier technique, regenerative endodontic treatment (RET) has been in the center of interest in recent years. This treatment protocol involves chemical disinfection of the root canal space, followed by the use of a triple antibiotic paste (TAP) comprised of metronidazole, ciprofloxacin and minocycline, as an intracanal medicament and the induction of bleeding to create a matrix for the ingrowth of new vital tissue in the pulp canal space [2]. Tooth discoloration is a major side effect that may be caused by minocycline component of the TAP [3]. Therefore, minocycline is omitted by some investigators, and ciprofloxacin and metronidazole are used in a double antibiotic paste (DAP). Most of the studies on regenerative endodontic procedures involve successful case reports and case series. As failures are usually not reported, it is not yet possible to predict the failure rate of RET. The failed cases where the regenerative endodontic procedure does not result in maturogenesis, require treatment with alternative techniques, such as the apical barrier technique. Various materials have been used in this technique but mineral trioxide aggregate (MTA) remains the material of choice for most dentists [4].

The sealing ability of MTA is a very important factor for successful endodontic treatment in open-apex teeth. The effects of pH, smear layer removal, MTA thickness, and calcium hydroxide paste on the marginal adaptation and sealing ability of MTA have been investigated in previous studies [5-9]. However, no study has been performed on the effect of DAT or TAP on the sealing ability of MTA. Therefore, the purpose of this study is to evaluate the effect of calcium hydroxide, DAP and TAP on the sealing ability of MTA.

## Materials and Methods

This study was approved by the Ethics Committee of Shiraz University of Medical Sciences (Grant no.: 94-01-03-9882). Ninety single-rooted, freshly extracted mature human teeth were used in this study. After removal of the periodontal tissue and calculus, the teeth were disinfected with 5.25% sodium hypochlorite (NaOCl) solution (Clorox, Oakland, CA, USA) for 30 min and then stored in distilled water. Teeth with extra canals, curved or calcified canals, resorption, or any signs of cracks were excluded from the study. The inclusion criteria were confirmed by mesiodistal and buccolingual radiographs and examination of the teeth under an operating microscope (Zeiss, Oberkochen, Germany). To eliminate root length as a variable, the crowns were sectioned so that all root segments were 18 mm long. A 3.0 mm slice of the root tip was resected using a straight fissure bur under water cooling. All specimens were instrumented with ProTaper Universal rotary files (Dentsply Maillefer, Ballaigues, Switzerland) up to F3 according to the manufacturer’s protocol to the working length. The irrigation was done by alternating use of 5 mL of 2.5% NaOCl and 5 mL of 17% EDTA (Pulpdent Corp., Watertown, MA, USA) between each instrument, using a 22-gauge needle. The standardized divergent open apex was created by means of a Peeso drill #1 to #6 (Mani, Tochigi, Japan) in a way that drill #6 was allowed to pass 1 mm beyond the apex. All procedures were carried out by a single operator.

Upon completion of instrumentation, the canals were dried with paper points (Diadent, Chongju City, Korea) and the specimens were randomly divided into four experimental groups (*n*=20) and two control groups (*n*=5). In group 1, TAP was applied by a lentulo spiral (Dentsply, Maillefer, Ballaigues, Switzerland) installed on low speed handpiece. In groups 2 and 3, DAP and calcium hydroxide paste, were applied in the same manner, respectively. In group 4, normal saline was used (normal saline group). The pastes were prepared with a powder to liquid ratio of 3:1 according to the study by Hoshino *et al.* [10] as follows: TAP group; equal amounts of metronidazole (Tehran Chimi, Tehran, Iran), ciprofloxacin (Iran Daru, Co. Tehran, Iran) and minocycline (Ratiopharm, Ulm, Germany) were mixed with distilled water, In DAP group; equal portions of metronidazole and ciprofloxacin were mixed with distilled water and in calcium hydroxide group; the calcium hydroxide paste was prepared by mixing calcium hydroxide powder (Golchay, Tehran, Iran) with distilled water.

The access cavities were filled with Cavisol (Golchay, Tehran, Iran). The apical portion of the specimens was covered by moistened gauze and all specimens were incubated at 37^°^C in 100% humidity for 3 weeks.

Afterwards, the temporary restoration was removed from the access cavity by a diamond fissure bur and all the medicaments were removed using #80 K-files (Mani, Tochigi, Japan) and irrigation with 0.5% NaOCl followed by 5 mL distilled water using a 27-gauge needle. Root canals were dried using standard #80 absorbent paper points (Diadent, Chongju City, Korea). A saline-moistened gauze pad was used to simulate periapical tissue to prevent over extrusion of MTA during condensation. 

ProRoot MTA (Tulsa Dental, Tulsa, OK, USA) was prepared according to the manufacturer’s recommendation. The material was placed through the access opening and condensed to the apical area just to the apex using a MTA carrier (Roydent Dental Products, Johnson City, TN, USA) and Schilder hand pluggers (Dentsply Caulk, Milford, DE, USA) in a 5 mm thickness. The density and thickness of MTA plugs were confirmed by radiographs. A moist pellet was placed on the MTA at 37^°^C for 48 h to allow initial setting. The MTA plug was then examined to ensure an acceptable set. Finger nail varnish was applied to the external tooth surface, excluding the resected root surface, to prevent side leakage through the dentinal tubules.

The negative and positive control groups were used to determine the efficacy of the microleakage measurement device. In the negative control group, after placing the MTA plug, two layers of nail varnish were applied over the entire surface of the root and MTA plug (*n*=5). In the positive control group the MTA plug was not placed (*n*=5).

**Figure 1:**
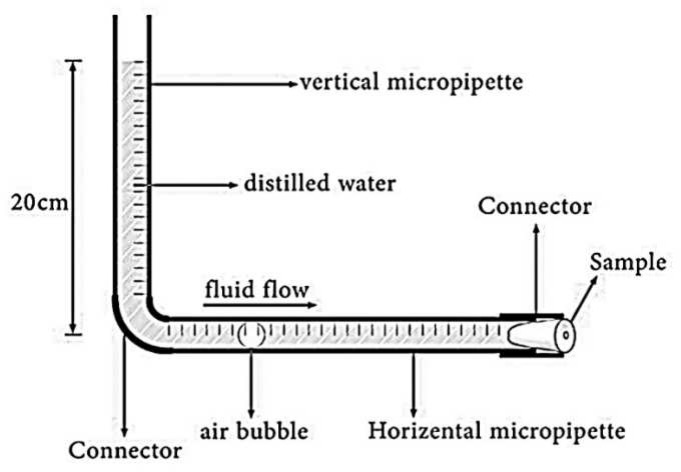
Fluid filtration measurement system. Schematic diagram of device used to measure microleakage

The effectiveness of MTA apical seal was evaluated using a fluid filtration technique with a continuous water pressure of 20 cm in days 1, 7, 14 and 30. This system evaluates fluid transport by measuring bubble movement. The measurement device was made by attaching two micropipettes perpendicular to each other. The apical part of each specimen was covered by cyanoacrylate glue (Interlock Co., LTD, Japan) and inserted in a piece of urine catheter. The free end of catheter was connected to the horizontal part of the device. The horizontal micropipette volume was 1 mL with 10 µL accuracy of measurement. The vertical micropipette was filled with saline up to 20 cm. A bubble was introduced into the horizontal micropipette using a microsyringe (Figure 1). The position of the air bubble was marked at the beginning and end of each day, and the linear air bubble movement on the micropipette was recorded in microliters. The height of the fluid in the horizontal micropipette was checked daily during the study and saline was added as necessary to maintain a height of 20 cm for all samples. The rate of microleakage in each day was calculated in µL/min/cm H_2_O according to following formula: Rate of microleakage=bubble displacement in each day (μL)/time of interval (min).

Data were analyzed by the repeated measures ANOVA test to evaluate the changes in sealing ability over time. One-way ANOVA/LSD was used to compare the mean rate of microleakage among the four groups at each time period.

## Results

No microleakage was observed in the negative control group after one month. Immediate fluid outflow was observed in the positive control group. All of the experimental groups exhibited different amounts of microleakage during the specified days, the results are presented in Table 1.

**Figure 2:**
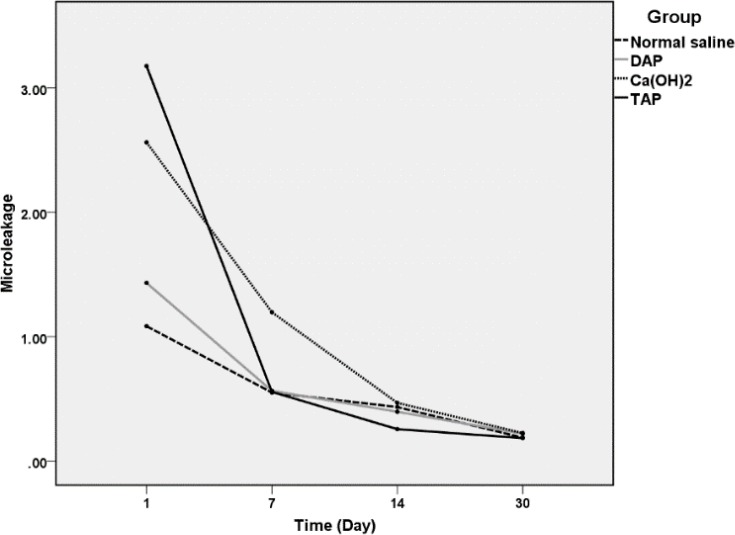
The rate of microleakage in experimental groups at different days (µL/min/cm H2O × 10

Repeated measures ANOVA showed a significant interaction effect between the experimental groups and times (*P*=0.001). In all study groups, microleakage decreased after one month (Figure 2). 

One way ANOVA showed that microleakage in the TAP group on day one was statistically greater than that of the DAP (*P*=0.024) and normal saline (*P*=0.04) groups. However, microleakage of calcium hydroxide group was not significantly different in comparison with that of other experimental groups (*P*>0.05). Moreover, the microleakage in the calcium hydroxide samples on day 7 was significantly greater than that of the other three experimental groups (*P*< 0.05). However, no significant difference was observed between any of the experimental groups on days 14 and 30 (*P*>0.05) (Table 1).

## Discussion

In this study, the effect of different intracanal pulp regeneration medicaments on the microleakage of MTA was investigated. The results showed that on day 1, the microleakage in the TAP group was significantly more than the calcium hydroxide, DAP or normal saline groups. However, on day 7 the rate of microleakage in the calcium hydroxide group was significantly more than the other three experimental groups. However, these differences disappeared in the third and fourth times and no significance differences were found at longer time points.

Various techniques have been used to measure the sealing ability of MTA, such as dye penetration, bacterial leakage, and fluid infiltration techniques. Dye penetration has some shortcomings, such as inconsistency in the depth of dye penetration due to different dye size and chemical reactivity. Trapped air in the root canal filling may also hinder dye penetration [11, 12]. Some studies have shown poor correlation between dye penetration and other techniques for assessing microleakage [13, 14]. Bacterial leakage is more clinically relevant than dye penetration. However, it also has limitations. For instance, usually only one strain of bacteria was used, as compared with the mixed flora found *in vivo* [15]. Therefore, the fluid filtration technique which was introduced by Pashley *et al.* [16] was used in this study, with some modifications. Unlike dye penetration and bacterial leakage, this method is quantitative and qualitative as well as non-destructive, thus microleakage can be measured for months on the same sample. Moreover, no alterations occur on samples during the procedures, such as immersing roots in acid, methyl salicylate or alcohol [14]. One limitation of this technique is the extreme variation of the applied pressure in published studies, so comparison between the results of different studies is difficult [14]. 

The thickness of MTA plug is variable in different studies. However, the best results have been reported with 3-5 mm thicknesses. Thus, 5-mm thickness was chosen for this study [5, 17]. Moreover, MTA without any gutta percha or sealer backpacking was used in order to eliminate any confounding factors.

Antibiotic pastes are important components in regenerative therapy and they have also been approved as more effective medicaments than calcium hydroxide paste in disinfection of canals and dentinal tubules [18, 19]. However, questions have been raised about the effect of these intracanal medicaments on the microleakage of MTA in the case of failed regenerative therapy or one visit apexification after using these medicaments. Changes in the microleakage of MTA can be attributed to the effects of medicaments on the chemical structure of root dentin, surface changes, and superficial collagen degradation and demineralization [20]. In addition, the acidic and alkaline pH of these medicaments may influence the sealing ability of apical plugs [6, 9].

In this study, the greater microleakage in the TAP group on day 1, might be related to the chelating action of minocycline, as minocycline binds to calcium ions and forms an insoluble complex [21]. Moreover, the remnant of this medicament may interfere with the setting of MTA and consequently influence its sealing ability. Berkhoff [22] pointed out that more than 80% of TAP could not be cleaned from the canal while calcium hydroxide was removed significantly better and the remaining paste was more superficial than TAP.

**Table 1.** The mean (SD) of microleakage (µL/min/cm H_2_O×10^-3^). Different letters in columns shows significance

**Time (day)**	**1**	**7**	**14**	**30**
**Normal saline**	1.08 (0.94) ^A^	0.55 (0.41) ^A^	0.43 (0.27) ^A^	0.19 (0.14) ^A^
**DAP**	1.43 (0.91) ^A^	0.56 (0.43) ^A^	0.4 (0.23) ^A^	0.22 (0.18) ^A^
**TAP**	3.17 (2.48) ^B^	0.56 (0.45) ^A^	0.26 (0.16) ^A^	0.18 (0.09) ^A^
**Calcium Hydroxide**	2.56(2.55) ^AB^	1.2 (0.68) ^B^	0.47 (0.34) ^A^	0.22 (0.11) ^A^

The influence of residual calcium hydroxide on the microleakage of MTA has been the subject of several studies with contradictory results. Stefopoulos *et al.* [23] found that calcium hydroxide adversely affects the sealing ability of a white MTA apical plug. However, the sealing ability of gray MTA was not influenced by calcium hydroxide pretreatment. They assumed that calcium hydroxide mechanically interferes or chemically reacts with MTA, and this chemical reaction may be more prominent in white MTA due to a different composition than gray MTA [23]. However, in a study by Bidar *et al.* [24] the effect of calcium hydroxide on short and long term sealing ability of MTA was investigated and no significant difference was found compared to control group. The difference in these findings in comparison with our results may be due to different methods of microleakage assessment, different duration of calcium hydroxide treatment, and different duration of microleakage assessment.

In our study, the rate of microleakage decreased in all of the experimental groups with the passage of the time, which is in agreement with previous relevant studies [9, 25-27]. This phenomenon can be ascribed to the hygroscopic expansion of MTA and filling of microscopic voids [28, 29]. Another hypothesis is the role of calcium carbonate production in the calcium hydroxide group, which could decrease microleakage [30]. This study evaluated MTA leakage using large and straight canals over a 1-month period of time. Long-term effects in canals that are small or curved should be evaluated. In addition, in a real clinical situation the irregular nature of open apices may limit the adaptation of root end filling material to the dentinal walls. One should also not overlook the fact that the fluid filtration technique has some innate variable factors such as the expansion and contraction of the polyethylene components in response to manipulation and thermal changes, which could affect the results. Therefore, the outcome of one visit apexification with MTA and other silicate based cement used as an apical barrier after using antibiotic pastes should be evaluated in future studies.

## Conclusion

According to the results of this *in vitro* study, the use of TAP and calcium hydroxide as intra-canal medicaments causes only a temporary effect on the microleakage of MTA plugs. All of the medicaments tested in this study can be used without any concern about permanent changes in the microleakage of MTA.
